# Factors Associated With Prosthesis Embodiment and Its Importance for Prosthetic Satisfaction in Lower Limb Amputees

**DOI:** 10.3389/fnbot.2020.604376

**Published:** 2021-01-15

**Authors:** Robin Bekrater-Bodmann

**Affiliations:** Department of Cognitive and Clinical Neuroscience, Central Institute of Mental Health, Medical Faculty Mannheim, Heidelberg University, Mannheim, Germany

**Keywords:** amputation—rehabilitation, human-machine interaction, prosthesis embodiment, prosthesis satisfaction, regression analysis

## Abstract

Perceptual integration of a prosthesis into an amputee's body representation, that is, prosthesis embodiment, has been proposed to be a major goal of prosthetic treatment, potentially contributing to the user's satisfaction with the device. However, insufficient knowledge about individual or prosthetic factors associated with prosthesis embodiment challenges basic as well as rehabilitation research. In the present study, hierarchical multiple regression analyses on prosthesis embodiment—as assessed with the recently introduced *Prosthesis Embodiment Scale*—were applied to the survey data of a large sample of prosthesis-using lower limb amputees, entering relevant objective-descriptive (i.e., unbiased characteristics of the amputation or the prosthesis) and subjective-evaluative variables (i.e., the amputee's perceptions related to the amputation or the prosthesis) as first- or second-level regressors, respectively. Significant regressors identified in these analyses together explained *R*^2^ = 36.3% of prosthesis embodiment variance in the present sample, with a lower level of amputation, less intense residual limb pain, more realistic visual appearance of the device, higher prosthetic mobility, and more positive valence of prosthesis-induced residual limb stimulations representing significantly associated factors. Using the identical set of regressors hierarchically complemented by prosthesis embodiment on measures of prosthetic satisfaction—as assessed with the *Trinity Amputation and Prosthesis Experience Scales*—revealed that prosthesis embodiment was significantly and positively associated with aesthetic as well as functional prosthesis satisfaction. These findings emphasize the importance of psychological factors for the integration of a prosthesis into the amputee's body representation, which itself represents a crucial factor associated with prosthesis satisfaction. The results might have important implications for future prosthetic treatment; however, replication of the findings in an independent sample is required, as well as sophisticated experimental designs in order to elucidate the causality of effects.

## Introduction

The amputation of a limb represents a severe impact on a person's physical integrity. The need to restore the body after amputation has been met with ever new prosthetic developments, seeking to equip the amputee with a virtually full replacement of the lost body part. To this end, prosthetic treatment is primarily guided by aspects of cosmetics and functionality.

Previous studies sought to reveal factors associated with the amputee's satisfaction related to the prosthetic device. Thus, better comfort of fit, better functionality, and more positive evaluation of weight (Glynn et al., 1986; Postema et al., 1999; Gallagher and MacLachlan, 2000; Biddiss and Chau, 2008; Sinha et al., 2014; Baars et al., 2018), higher frequency of prosthesis use (Dillingham et al., 2001), and more preferable appearance of the device (Postema et al., 1999; Harness and Pinzur, 2001) have been reported to be associated with higher prosthesis satisfaction or reduced prosthesis rejection in both upper and lower amputees. Individual characteristics of the amputation have also been identified as co-varying with prosthesis satisfaction, with a lower (compared to higher) amputation level, better residual limb health, and less post-amputation pain being often reported factors (Harness and Pinzur, 2001; Biddiss and Chau, 2007; Desmond et al., 2008; Berke et al., 2010; Webster et al., 2012; Sinha et al., 2014; Resnik et al., 2020).

A recent systematic review on factors associated with prosthesis satisfaction specifically in lower limb amputees (Baars et al., 2018) identified the device's appearance, functional and physical properties, and fit, as well as prosthesis use and medical issues of the residual limb as important variables. Sex, etiology and the level of amputation, as well as properties of the prosthesis socket might represent crucial modulating variables.

As clinically relevant as these results might be, they neglect the importance of psychological factors, such as the amputees' perceptions of their body in relation to their prosthesis. Recently, particular interest has been taken in the mechanisms underlying the so-called *prosthesis embodiment*, which describes the integration of the prosthetic device into the amputee's body representation (Murray, 2004, 2008; Makin et al., 2017; Niedernhuber et al., 2018). Prosthesis embodiment could be associated with several positive rehabilitative outcomes. Thus, embodied upper limb prostheses have recently been related to a stabilized body posture (Imaizumi et al., 2016) and improved motor planning (Gouzien et al., 2017). Furthermore, prosthesis embodiment has been epidemiologically related to reduced levels of post-amputation pain in both upper and lower limb amputees (Kern et al., 2009; Bekrater-Bodmann et al., 2020). Other results revealed prosthesis use-dependent brain plasticity, suggesting neural embodiment of the device (Lotze et al., 1999; Maimon-Mor and Makin, 2020). Psychometrically, prosthesis embodiment experiences in lower limb amputees have recently been characterized by (a) the sensation that the device is an actual body part, (b) the feeling of an intact physical integrity, (c) the experience of the prosthesis' posture and location as anatomically plausible, and (d) having control over the device's movements (Bekrater-Bodmann, 2020). In other words, prosthesis embodiment is the cognitive and perceptual incorporation of the prosthesis, which can then be better described as representing an actual body part, rather than as a mere tool loosely attached to the body (Murray, 2004; Makin et al., 2017). Recent results suggest a wide range in the intra-individual degree of prosthesis embodiment experiences in lower limb amputees, which have been shown to represent a temporally stable, but contextually dynamic perceptual feature co-varying with a given prosthetic device (Bekrater-Bodmann, 2020).

Research on factors associated with the embodiment of a prosthesis is scarce. Epidemiological studies on samples of both upper and lower limb amputees reported associations between certain manifestations of prosthesis embodiment and the presence of non-painful phantom phenomena (Giummarra et al., 2010), the absence of phantom limb pain (Kern et al., 2009), as well as a younger age, a leg (vs. arm) amputation, a longer residual limb, a longer time since amputation, and the frequency of prosthesis use (Bekrater-Bodmann et al., 2020). However, the interpretation of these results is complicated by heterogeneous operationalizations of prosthesis embodiment experiences with unknown validity. With the recently introduced *Prosthesis Embodiment Scale* (PEmbS; Bekrater-Bodmann, 2020), however, a psychometrically evaluated instrument for the assessment of prosthesis embodiment experiences is available, which might help to reliably identify factors associated with the perceptual integration of the prosthesis into an amputee's body representation. Moreover, since there is evidence for complex inter-relationships between individual and prosthesis variables (Bekrater-Bodmann et al., 2020), the often-used univariate statistical approaches (such as correlations for continuous data or tests for comparing the central tendency in subgroups) might be inappropriate to validly identify factors associated with prosthesis embodiment. Thus, multivariate statistical approaches are needed, which along with homogenous samples of sufficient sizes might help to identify factors associated with perceptual prosthesis embodiment. Knowledge about these factors might have important implications for theoretical concepts on body perception as well as future prosthetic design and treatment.

In the present study, a regression analytical approach was used, including a large sample of unilateral lower limb amputees using a prosthesis. Amputation and prosthesis factors were selected based on empirical results on prosthesis embodiment and prosthesis satisfaction. For both amputation and prosthesis factors, hierarchical models were applied, including objective-descriptive (i.e., unbiased characteristics of the amputation or the prosthesis) and subjective-evaluative variables (i.e., the amputee's perceptions related to the amputation or the prosthesis) as first- or second-level regressors, respectively. Regressors identified to be significantly associated with prosthesis embodiment were combined in order to better explain the variance in the criterion. Finally, the importance of prosthesis embodiment for prosthesis satisfaction was explored.

## Materials and Methods

### Sample and Procedure

Between May 2019 and March 2020, 166 unilateral lower limb amputees using a prosthesis were recruited (71.69% male; mean age of 56.63 years with a standard deviation of 10.95). Various sources were used for recruitment, such as a previously established data base (initial description by Bekrater-Bodmann et al., 2015), flyers distributed to professionals working in prosthetic rehabilitation centers, and calls via print and social media. Inclusion criteria for the present study were an age between 18 and 80 years, unilateral lower limb loss, owning and using a prosthesis, and sufficient comprehension of the German language. The sample consisted of 118 participants, which were already described in a recent study on prosthesis embodiment (Bekrater-Bodmann, 2020), augmented by 48 newly recruited lower limb amputees. Clinical details of the present sample are provided in Table 1.

**Table 1:** Amputation details of the present sample (*N* = 166).

**Characteristics**	**%**
**Amputation**	
Amputation of dominant limb^a^	37.25
Amputation of left limb	61.45
**Level of amputation**	
Foot amputation	3.01
Transtibial amputation	46.99
Knee exarticulation^b^	4.82
Transfemoral amputation	44.58
Hip exarticulation or hemipelvectomy	0.60
**Reason for amputation (multiple responses allowed)**	
Accident	63.86
Cancer	18.07
Injury	15.66
Infection	12.65
Peripheral vascular disease	8.43
Congenital limb deficiency	0.60
Other reasons	9.04

a *valid data for the question Which leg did you use to kick an object, for example a ball, prior to amputation? (13 missing data due to not-remembering)*;

b *one participant with rotationplasty was assigned to this category*.

Recruited amputees were screened via telephone interviews for eligibility to participate before the consent form was sent postally or via email. After returning the consent form, an individualized link to an online questionnaire battery (implemented in Gorilla™; https://gorilla.sc/) was sent to most of the participants. Due to not having access to the internet, a printed version of the questionnaire battery was sent to ten participants postally. The battery (online or print version) included questionnaires on demographics, amputation, and prosthesis information, phantom phenomena, as well as instruments on mobility, prosthesis acceptance, psychosocial functioning, and body image. The study was approved by the ethics review board of the Medical Faculty Mannheim, Heidelberg University, and adhered to the Declaration of Helsinki in its current form.

### Selection of Factors

Since it has previously been argued that prosthesis embodiment could contribute to prosthesis satisfaction (MacLachlan, 2004; Murray, 2008), the selection of regressors was guided by empirical results on both phenomena as identified in lower limb amputees. A recent systematic review on factors associated with prosthesis satisfaction in lower limb amputees (Baars et al., 2018) identified appearance, fit, and use of the prosthesis, medical issues of the residual limb, as well as properties of the device as important influencing factors, with sex, etiology of amputation, properties of the prosthesis socket, and the level of amputation representing crucial modulating variables. Further, Bekrater-Bodmann et al. (2020) found that a younger age, a longer residual limb, an increased amount of time since amputation, a higher frequency of prosthesis use, and the type of prosthesis (modular vs. exoskeletal) were associated with higher prosthesis ownership—representing a sub component of embodiment (Longo et al., 2008)—in a sample of more than 1,300 lower limb amputees. Phantom and residual limb sensations have further been related to prosthesis embodiment (Kern et al., 2009; Giummarra et al., 2010; Bekrater-Bodmann et al., 2020). Most of these variables can be categorized as being related to the amputation or the prosthesis, and can further be described as being objective-descriptive (i.e., unbiased characteristics of the amputation or the prosthesis) or subjective-evaluative features (i.e., the amputee's perceptions related to the amputation or the prosthesis). The grouping as well as operationalization of the variables is described below. Since there is evidence that surveyed amputees are rather unable to reliably indicate technical specifications of their prosthetic device (Bekrater-Bodmann, 2020), purely technical properties of the prosthesis were not included as regressors in the present study.

### Operationalization and Grouping of Factors

The operationalization of included factors, grouped by content, is described below. Additional information, including the wording (translated to English) and the scaling of used items as well as the variables derived from them, is provided in Supplementary Table 1.

#### Amputation-Related Factors

##### Objective-Descriptive Amputation-Related Factors (First-Level Regressors)

As reviewed above, the level and etiology of and the time since amputation might influence prosthesis embodiment and/or prosthesis satisfaction in lower limb amputees. *Time since amputation* was measured in years, by using the difference between the date of amputation and the date of participation in the present study.

Since the majority of the included amputees in the present study suffered from transtibial or transfemoral amputation, both with a percentage of about 45% (see Table 1), the *level of amputation* was dichotomized and dummy-coded with 0 (foot and transtibial amputation) for *low amputation level* and 1 (knee exarticulation, transfemoral amputation, and hip exarticulation or hemipelvectomy) for *high amputation level*).

Most participants reported traumatic events such as accidents or injuries as the reason for their amputation; a minority reported other reasons such as cancer or peripheral vascular disease, and about 20% of the participants indicated more than one reason. *Etiology of amputation* was thus dichotomized into one group that indicated traumatic events (i.e., accidents or injuries) as reason for amputation (dummy-coded as 0), and another group that reported other or multiple reasons for the amputation (dummy-coded as 1; cf., Table 1).

##### Subjective-Evaluative Amputation-Related Factors (Second-Level Regressors)

Painful or non-painful sensations in the residual and phantom limb are the most common perceptual consequences following a limb amputation (e.g., Ehde et al., 2000), and there is evidence for different etiologies of these sensations (e.g., Foell et al., 2011). In the present study, three different phenomena were considered: phantom limb awareness (PLA), that is, the perceived presence of the amputated limb; phantom limb pain (PLP), that is, painful experiences located in the missing limb; and residual limb pain (RLP), that is, painful experiences located in the remaining part of the amputated limb.

Participants were separately asked whether they had experienced PLA, PLP, and RLP in the past three months (i.e., current presence of the phenomenon). Participants who responded affirmatively were then asked to indicate the average intensity of the phenomena in the past 4 weeks, using a numerical rating scale ranging from 0 (no pain/no sensations) to 10 (unbearable pain/very strong sensations). This measure was used as *PLA intensity, PLP intensity*, and *RLP intensity*, respectively.

#### Prosthesis-Related Factors

##### Objective-Descriptive Prosthesis-Related Factors (First-Level Regressors)

Participants were instructed to answer all prosthesis-related questions for their main prosthesis, which—in the case of owning more than one prosthesis—is the prosthesis they use most of the time.

It has been proposed that the perceptual system of amputees can change over time (Ehrsson et al., 2008) which would not only be true for time since amputation, but also for the time the amputee is faced with a given prosthetic device. Thus, participants were asked to indicate since when they had been using their current prosthesis, and *time with current prosthesis* was calculated in years.

Further, participants were asked how often they use the prosthesis (a) per week (1—less than twice; 2—every 2nd day; 3—almost daily; 4—daily) and (b) per day (1−1–2 h; 2—several hours, but not throughout; 3—half a day; 4—from morning to evening). By multiplying both ratings, an ordinally scaled *frequency of prosthesis use* index with nine ranks was obtained, ranging from rare to frequent use (cf., Bekrater-Bodmann et al., 2020).

##### Subjective-Evaluative Prosthesis-Related Factors (Second-Level Regressors)

Selection of subjective-evaluative prosthesis-related factors based on cosmetics, functionality, and fitting of the device, and thus included perceptions and evaluations of these aspects. Thus, participants were asked to judge the visual appearance of their prosthesis regarding resemblance with a real body part using a numerical rating scale ranging from 0 (artificial) to 10 (like an actual body part), assessing *visual realism* of the device. This feature of appearance was chosen (cf., Baars et al., 2018), since there is empirical evidence that visual realism can facilitate the experimental induction of embodiment experiences (Tsakiris et al., 2010).

*Mobility* was assessed using the German 12-item version of the Prosthetic Limb Users Survey of Mobility (PLUS-M-12; Hafner et al., 2017), a self-report instrument assessing mobility of lower limb amputees. The PLUS-M-12 asks for the perceived ability to perform given everyday actions (e.g., “Are you able to walk a short distance in your home?”), using a response scale ranging from 0 (without any difficulty) to 4 (unable to do). Raw sum scores were converted to *T*-values, representing a standardized score with a mean of 50 and an SD of 10 (according to the guidelines; University of Washington Center on Outcomes Research in Rehabilitation, 2014). For the purpose of intuition, the scores were reversed so that a higher score indicates a higher level of mobility.

Finally, participants were asked whether or not the prosthesis caused stimulations at the stump and if so, how these stimulations were evaluated, using a Likert scale ranging from −5 (negative) to +5 (positive). For this *residual limb stimulation* measure, negative ratings were recoded to −1, neutral (i.e., 0) or absent stimulations to 0, and positive ratings to +1.

### Operationalization of Criterion Variables

#### Prosthesis Embodiment

The *Prosthesis Embodiment Scale for Lower Limb Amputees* (PEmbS-LLA; Bekrater-Bodmann, 2020) consists of ten items targeting the dimensions of *Ownership/Integrity* (a sense of belongingness for the prosthesis), *Agency* (a sense of being in control of the prosthesis), and *Anatomical Plausibility* (referring to spatial-structural properties of the prosthesis in respect to the amputee's body). These dimensions correspond to the earlier identified embodiment components in the rubber hand illusion paradigm (*Ownership, Agency*, and *Location*; Longo et al., 2008), and thus quantify the degree to which a prosthesis is cognitively and perceptually integrated as a part of the amputee's body, rather than a mere tool (cf., Makin et al., 2017). Participants were asked to look at or walk with the prosthesis before indicating their agreement or disagreement with given statements (for example “The prosthesis is part of my body”), using a Likert scale ranging from −3 (strongly disagree) to +3 (strongly agree). The total score of the PEmbS-LLA, representing an overall measure of perceived prosthesis embodiment, was calculated by averaging all valid items (up to one missing item was allowed in the present study, which was the case in *n* = 4 participants; another four participants had more than one missing value or were not able to walk with their prosthesis and were thus excluded from the subsequent analyses), with higher scores indicating higher prosthesis embodiment. The *Prosthesis Embodiment Scale* has previously been shown to have good to excellent reliability (Bekrater-Bodmann, 2020), and results on implicit effects suggest validity of the instrument (Fritsch et al., 2020).

#### Prosthesis Satisfaction

Prosthesis satisfaction was assessed with a German translation of the *Trinity Amputation and Prosthesis Experience Scales—Revised* (TAPES-R; Gallagher et al., 2010), provided by the Center for Orthopedic and Trauma Surgery, Heidelberg University Hospital, Heidelberg, Germany. The TAPES-R satisfaction sub-scale measures two dimensions of prosthesis satisfaction: *aesthetic prosthesis satisfaction* is measured with three items targeting the satisfaction level with color, shape, and appearance of the device; and *functional prosthesis satisfaction* is operationalized by five items focusing on satisfaction with weight, usefulness, reliability, fit, and comfort. Each item was answered using the response scale 1 (not satisfied), 2 (satisfied), and 3 (very satisfied). In order to enhance comparability of scores, the means of the items representing a scale (in contrast to summing them up) were calculated (ranging from 1 to 3 each).

### Statistical Analysis

All analyses were performed with IBM SPSS v26. First, descriptive analyses of the included variables were performed, and prevalence, means, standard deviations (SD), medians, and/or interquartile ranges are provided, based on the scaling level.

In order to check whether the statistical assumptions for performing regression analyses were fulfilled, the author initially checked (a) for violation of the residuals' normal distribution using Shapiro–Wilk tests, (b) the absence of multicollinearity, which was assumed if tolerances >0.20 and variance inflation factors (*VIF*) < 4.0 (Hair et al., 2010), (c) the absence of heteroscedasticity using the Breusch-Pagan test (given that kurtosis of all residual distributions was < ±2; George and Mallery, 2010), (d) absence of auto correlations (checking visually the Q-Q plot; Golberg and Cho, 2010), and (e) absence of endogeneity (all correlations between residuals and regressors *r* < 0.001, all *p* > 0.999). Each model (described below) fulfilled the assumptions; statistical details are provided in the **Supplement**.

**Figure 1:**
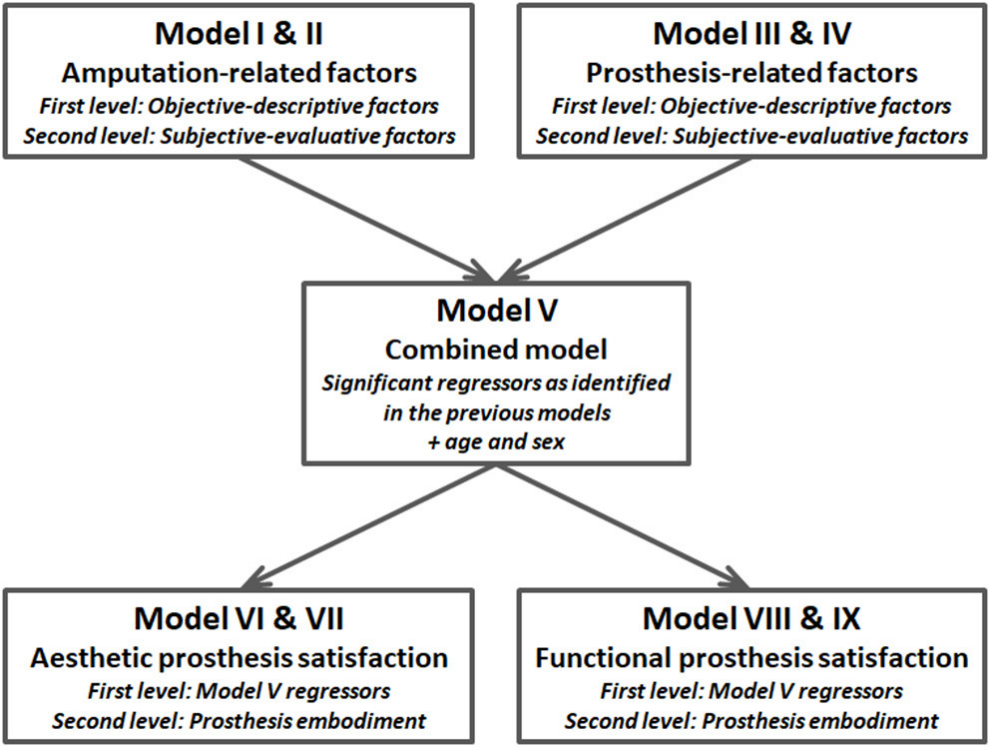
Block diagram visualizing the analytic strategy. Regressors are described in detail in the main text.

In order to test for associations between prosthesis embodiment and amputation- and prosthesis-related factors, hierarchical multiple regression analyses were performed. Separately for amputation- (models I and II) and prosthesis-related factors (models III and IV), first-level (objective-descriptive) and second-level (subjective-evaluative) regressors were entered block-wise (simultaneous entry). The regressors which emerged to be significant in models II and IV were then simultaneously entered in model V along with the demographic variables *sex* and *age*. Finally, the model-V-set of regressors was used to explain variance in aesthetic (model VI) and functional prosthesis satisfaction (model VIII). In a second hierarchical level each, prosthesis embodiment was entered (models VII and IX). The analytic strategy is visualized in Figure 1. For each model, the analysis of variance testing for significance of *R*^2^ and/or its increase in hierarchical models is reported. Further, the author reports on the adjusted *R*^2^. For each regressor, the unstandardized coefficient *B* and its standard error *SE* were reported, along with the standardized regression coefficient β and the respective *p*-value. Note that the number of participants included in the regression analyses varies between *n* = 159 and 161, depending on the availability of valid data (cf., Table 2).

**Table 2:** Descriptive details of the included variables (*N* = 166).

**Variables**	***M* (*SD*)**	***Mdn* (*IQR*)**	**%**	***n* missing data**
**Objective-descriptive amputation-related variables**				
Level of amputation (high/low)	–	–	50.00/50.00	0
Time since amputation (years)	27.35 (16.11)	–	–	0
Etiology of amputation (traumatic / other reasons or combinations)	–	–	56.02/43.98	0
**Subjective-evaluative amputation-related variables**				
PLA intensity (0–10)	3.06 (3.48)	–	–	1
PLP intensity (0–10)	2.95 (3.44)	–	–	0
RLP intensity (0–10)	1.41 (2.49)	–	–	0
**Objective-descriptive prosthesis-related variables**				
Time with current prosthesis (years)	4.14 (5.72)	–	–	0
Frequency of prosthesis use (rank 1–9)	–	9.00 (0.00)	–	2
**Subjective-evaluative prosthesis-related variables**				
Visual realism (0–10)	4.10 (3.02)	4.00 (5.00)	–	0
Mobility (reversed *T* scores)	59.12 (8.17)	60.00 (9.70)	–	1
Residual limb stimulation (−1—negative/0—neutral or absent/+1—positive)	–	0.00 (1.00)	40.96/43.37/15.66	0
**Criterion variables**				
Prosthesis embodiment (non-transformed; −3 to +3)	1.44 (1.16)	1.60 (1.30)	–	4
Prosthesis embodiment (transformed; 0 to √6, i.e., ≈2.45)	1.31 (0.49)	1.27 (0.57)	–	4
Aesthetic prosthesis satisfaction (1–3)	2.25 (0.48)	2.00 (0.67)	–	0
Functional prosthesis satisfaction (1–3)	2.28 (0.47)	2.20 (0.60)	–	0

*PLA, phantom limb awareness; PLP, phantom limb pain; RLP, residual limb pain; M, mean; SD, standard deviation; Mdn, median; IQR, interquartile range; N / n = number*.

Since the PEmbS-LLA total score distribution differed significantly from normality (Shapiro–Wilk test, *W*_162_ = 0.92, *p* < 0.001) and was characterized by positive skewness, reverse square root transformation was applied to positivize values (re-reversed score ranged from 0 to √6, i.e., ≈2.45, with higher values still describing higher prosthesis embodiment), which normalized data distribution (*W*_162_ = 0.99, *p* = 0.13). For the subsequent regression analyses, this normalized transformed score was used. Note that the initial use of the non-transformed PEmbS-LLA total score as criterion variable in the regression analyses for models I–IV resulted in residuals significantly differing from normal distribution (*W*_159−161_ = 0.94–0.97, all *p* ≤ 0.003), which represents a violation of requirements for the use of multiple regression analyses (see above). This was avoided by the use of the transformed PEmbS-LLA score (for the respective statistics, see the **Supplement**). For aesthetic and functional prosthesis satisfaction as assessed with the TAPES-R, residual distribution was normal and all other assumptions required for regression analyses were fulfilled (see **Supplement**), so that no transformation procedure was applied to these data.

## Results

### Descriptive Analyses

The descriptive details for the used variables can be found in Table 2. High and low amputation levels were equally distributed, and the amputation dated back more than 25 years on average (range: 0–72 years). About 56% of amputees indicated a traumatic event as the only reason for their amputation. Prevalence of PLA, PLP, and RLP in the last three months was 64.46, 53.01, and 31.33%, respectively (no missing data). Mean intensity in the last 4 weeks in the whole sample was low to medium; in those participants who reported the respective phenomenon, however, mean intensity was 6.01 (SD = 2.45) for PLA, 5.63 (SD = 2.73) for PLP, and 4.68 (SD = 2.31) for RLP, representing medium levels. *Time with current prosthesis* averaged more than 4 years, with a wide range in the individuals from 0 to 34 years. *Frequency of prosthesis use* was high, which is expectable for lower limb amputees (Raichle et al., 2008). With an average rating of about 4, *visual realism* was rated medium. Participants rated *mobility* with their device as high (compared to norm data from a representative sample of lower limb amputees; cf., University of Washington Center on Outcomes Research in Rehabilitation, 2014). About 41% of the sample stated that the prosthesis caused negative stimulations on their residual limb, and more than 43% described these stimulations as neutral or being absent. A minority of about 16% described the residual limb stimulations as positive. About 87% of participants reported some degree of prosthesis embodiment (PEmbS-LLA score > 0, non-transformed data). With an average rating of about 2.3 each, aesthetic and functional prosthesis satisfaction was high in the present sample.

### Multiple Regression Analyses

#### Hierarchical Regression Analyses for Amputation- and Prosthesis-Related Factors

##### Hierarchical Regression Analysis for Amputation-Related Factors (Models I and II)

Model I, entering objective-descriptive amputation-related factors, was characterized by a significant determination coefficient [*F*_(3,157)_ = 3.483, *p* = 0.017], with *level of amputation* emerging as significant regressor. This model explained *R*^2^ = 6.2% of variance of prosthesis embodiment. Model II, adding subjective-evaluative amputation-related factors also was significant [*F*_(6,154)_ = 4.495, *p* < 0.001], with *level of amputation* and *RLP intensity* as individual significant regressors. This indicates that a lower amputation level, i.e., a longer residual limb, and less severe residual limb pain are significantly associated with prosthesis embodiment. Model II explained in total *R*^2^ = 14.9% of prosthesis embodiment variance, with the increase in the determination coefficient from model I to model II being significant [*F*_(3,154)_ = 5.225, *p* = 0.002]. Details of the analysis are given in Table 3.

**Table 3:** Hierarchical regression analysis (simultaneous entry of regressors) on prosthesis embodiment with objective-descriptive (model I) and objective-descriptive + subjective-evaluative amputation-related factors (model II) in *n* = 161 lower limb amputees.

**Model**	**Regressors**	***B***	***SE***	**β**	***p***	***R*^**2**^**	**Adjusted *R*^**2**^**	***p* for *R*^**2**^**	***p* for *R*^**2**^ increase by including subjective-evaluative regressors**
I— Objective-descriptive amputation-related regressors	(Constant)	1.396	0.107		<0.001	0.062	0.044	0.017	–
	Level of amputation^a^	−0.198	0.078	−0.202	0.012				
	Time since amputation	0.002	0.003	0.056	0.507				
	Etiology of amputation^b^	−0.073	0.085	−0.074	0.395				
II— Objective-descriptive + subjective-evaluative amputation-related regressors	(Constant)	1.533	0.115		<0.001	0.149	0.116	<0.001	0.002
	Level of amputation^a^	−0.198	0.077	−0.201	0.011				
	Time since amputation	0.001	0.003	0.020	0.809				
	Etiology of amputation^b^	−0.075	0.082	−0.076	0.363				
	PLA intensity	<0.001	0.012	−0.001	0.992				
	PLP intensity	−0.009	0.013	−0.065	0.456				
	RLP intensity	−0.055	0.015	−0.279	<0.001				

a *0, low; 1, high*;

b *0, accidents or injuries; 1, other reasons or combinations; PLA, phantom limb awareness; PLP, phantom limb pain; RLP, residual limb pain*.

##### Hierarchical Regression Analysis for Prosthesis-Related Factors (Models III and IV)

Model III, including objective-descriptive prosthesis-related factors, had a significant determination coefficient of *R*^2^ = 5.3% [*F*_(2,156)_ = 4.348, *p* = 0.015], with *frequency of prosthesis use* emerging as a significant regressor, indicating that the more often the prosthesis is actively used, the higher is the perceived embodiment for the prosthesis. The determination coefficient of model IV, to which subjective-evaluative prosthesis-related regressors were added, again was highly significant [*F*_(5,153)_ = 14.713, *p* < 0.001], with *visual realism, mobility*, and *residual limb stimulation* being significant regressors. This indicates that a higher degree of the prosthesis' visual similarity to a real limb, higher levels of prosthesis functionality, and the absence of negatively perceived stimulation caused by the prosthesis are positively related to prosthesis embodiment. The initially identified regressor *frequency of prosthesis use*, however, did no longer emerge as being significant in this extended model. In total, model IV explained *R*^2^ = 32.5% of the variance in prosthesis embodiment, with the increase in the determination coefficient from model III to model IV being highly significant [*F*_(3,153)_ = 20.534, *p* < 0.001]. Details of these analyses are given in Table 4.

**Table 4:** Hierarchical regression analysis (simultaneous entering of regressors) on prosthesis embodiment with objective-descriptive (model III) and objective-descriptive + subjective-evaluative prosthesis-related factors (model IV) in *n* = 159 lower limb amputees.

**Model**	**Regressors**	***B***	***SE***	**β**	***p***	***R*^**2**^**	**adjusted *R*^**2**^**	***p* for *R*^**2**^**	***p* for *R*^**2**^ increase by including subjective-evaluative regressors**
III—Objective-descriptive prosthesis-related regressors	(Constant)	0.589	0.246		0.018	0.053	0.041	0.015	–
	Time with current prosthesis	0.003	0.007	0.035	0.658				
	Prosthesis use frequency	0.082	0.028	0.229	0.004				
IV—Objective-descriptive + subjective-evaluative prosthesis-related regressors	(Constant)	−0.507	0.304		0.097	0.325	0.303	<0.001	<0.001
	Time with current prosthesis	−0.005	0.006	−0.056	0.409				
	Prosthesis use frequency	0.044	0.025	0.122	0.076				
	Visual realism	0.053	0.011	0.332	<0.001				
	Mobility	0.021	0.004	0.334	<0.001				
	Residual limb stimulation^a^	0.145	0.046	0.210	0.002				

a *-1, negative; 0, neutral or absent; +1, positive*.

#### Combined Regression Analysis (Model V)

For the combined regression analysis, the regressors identified to individually explain prosthesis embodiment in the previous analyses (i.e., *level of amputation, RLP intensity, visual realism, mobility*, and *residual limb stimulation*) were entered simultaneously, along with *sex* and *age*. The model's determination coefficient of *R*^2^ = 36.3% was highly significant [*F*_(7,153)_ = 12.430, *p* < 0.001], with all entered variables emerging as individual regressors for prosthesis embodiment, each in the previously described direction. *Sex* and *age* were not significantly associated with prosthesis embodiment. Details of this analysis are provided in Table 5.

**Table 5:** Regression analysis (model V, simultaneous entering of regressors) on prosthesis embodiment with identified amputation- and prosthesis-related factors, controlling for sex and age, in *n* = 161 lower limb amputees.

**Regressors**	***B***	***SE***	**β**	***p***	***R*^**2**^**	**Adjusted *R*^**2**^**	***p* for *R*^**2**^**
(constant)	−0.015	0.360		0.967	0.363	0.333	<0.001
Sex^a^	−0.103	0.078	−0.095	0.189			
Age	0.002	0.003	0.047	0.505			
Level of amputation^b^	−0.139	0.068	−0.142	0.042			
RLP intensity	−0.038	0.013	−0.190	0.005			
Visual realism	0.048	0.011	0.297	<0.001			
Mobility	0.021	0.005	0.325	<0.001			
Residual limb stimulation^c^	0.137	0.046	0.198	0.003			

a *0, female; 1, male*;

b *0, low; 1, high*;

c *-1, negative; 0, neutral or absent; +1, positive; RLP, residual limb pain*.

#### Hierarchical Regression Analyses on Prosthesis Satisfaction (Models VI–IX)

For the hierarchical regression analyses on aesthetic (for statistical details see Table 6A) and functional prosthesis satisfaction (for statistical details see Table 6B), the regressors used in model V were entered (first-level regressors, models VI and VIII) and hierarchically complemented by prosthesis embodiment (second-level regressors, models VII and IX; see Figure 1).

**Table 6:** Hierarchical regression analysis (simultaneous entering of regressors) on aesthetic prosthesis satisfaction **(A)** and functional prosthesis satisfaction **(B)**, with identified factors (model VI and VIII, respectively) and additionally added prosthesis embodiment (model VII and IX, respectively), controlling for sex and age, in *n* = 161 lower limb amputees.

**Model**	**Regressors**	***B***	***SE***	**β**	***p***	***R*^**2**^**	**adjusted *R*^**2**^**	***p* for *R*^**2**^**	***p* for *R*^**2**^ increase by including prosthesis embodiment**
**(A)—criterion: aesthetic prosthesis satisfaction**									
VI— Identified regressors	(Constant)	2.404	0.409		<0.001	0.143	0.104	0.001	–
	Sex^a^	0.234	0.089	0.220	0.009				
	Age	−0.008	0.004	−0.181	0.027				
	Level of amputation^b^	−0.038	0.077	−0.039	0.627				
	RLP intensity	−0.027	0.015	−0.140	0.072				
	Visual realism	0.034	0.012	0.217	0.006				
	Mobility	0.001	0.005	0.015	0.865				
	Residual limb stimulation^c^	0.072	0.052	0.106	0.171				
VII— Identified regressors + prosthesis embodiment	(Constant)	2.408	0.400		<0.001	0.186	0.144	<0.001	0.005
	Sex^a^	0.260	0.087	0.244	0.003				
	Age	−0.008	0.003	−0.193	0.016				
	Level of amputation^b^	−0.002	0.076	−0.002	0.978				
	RLP intensity	−0.018	0.015	−0.091	0.224				
	Visual realism	0.022	0.013	0.140	0.084				
	Mobility	−0.004	0.006	−0.070	0.433				
	Residual limb stimulation^c^	0.037	0.052	0.054	0.485				
	Prosthesis embodiment	0.255	0.090	0.260	0.005				
**(B)—criterion: functional prosthesis satisfaction**									
VIII— Identified regressors	(Constant)	1.596	0.386		<0.001	0.181	0.143	<0.001	–
	Sex^a^	0.090	0.084	0.087	0.285				
	Age	−0.001	0.003	−0.032	0.686				
	Level of amputation^b^	−0.072	0.073	−0.078	0.321				
	RLP intensity	−0.040	0.014	−0.214	0.005				
	Visual realism	0.018	0.011	0.117	0.124				
	Mobility	0.012	0.005	0.202	0.017				
	Residual limb stimulation^c^	0.086	0.049	0.132	0.082				
IX— Identified regressors + prosthesis embodiment	(Constant)	1.601	0.373		<0.001	0.239	0.199	<0.001	0.001
	Sex^a^	0.119	0.081	0.116	0.145				
	Age	−0.002	0.003	−0.046	0.548				
	Level of amputation^b^	−0.033	0.071	−0.035	0.647				
	RLP intensity	−0.029	0.014	−0.157	0.039				
	Visual realism	0.004	0.012	0.027	0.728				
	Mobility	0.006	0.005	0.104	0.228				
	Residual limb stimulation^c^	0.047	0.049	0.072	0.339				
	Prosthesis embodiment	0.285	0.084	0.302	0.001				

a *0, female; 1, male*;

b *0, low; 1, high*;

c *-1, negative; 0, neutral or absent; +1, positive; RLP, residual limb pain*.

**Figure 2:**
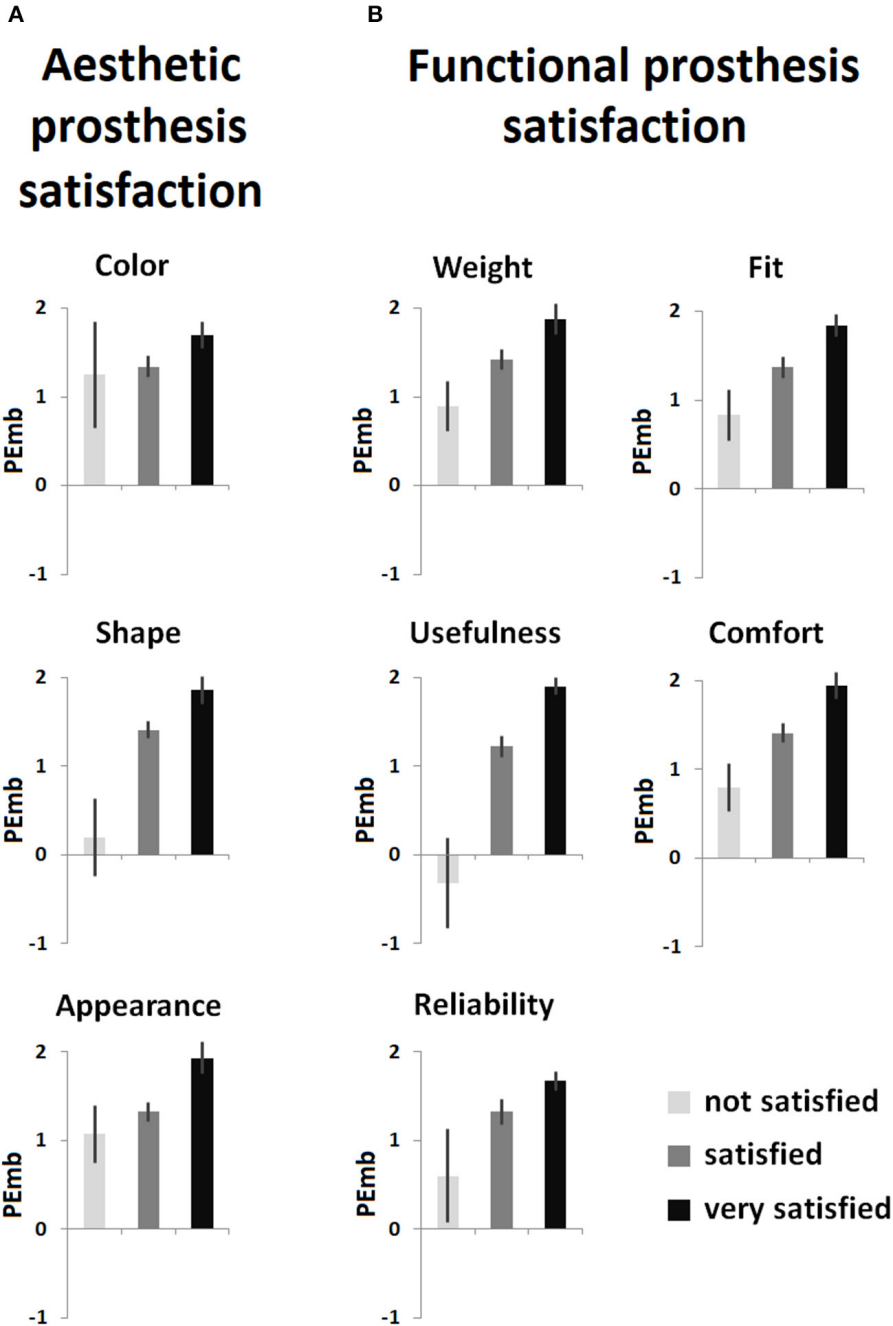
Item-wise relationship between levels of prosthesis satisfaction as measured with the prosthesis satisfaction sub-scales of Trinity Amputation and Prosthesis Experience Scales—Revised questionnaire and prosthesis embodiment (PEmb) as measured with the Prosthesis Embodiment Scale (non-transformed scores with a potential range from −3 to +3). **(A)** Aesthetic prosthesis satisfaction; **(B)** functional prosthesis satisfaction. Given are the mean values; error bars indicate the standard error of the mean.

Model VI was characterized by a significant determination coefficient [*F*_(7,153)_ = 3.658, *p* = 0.001], with *sex, age*, and *visual realism* significantly regressing on aesthetic prosthesis satisfaction. This indicates that the younger the prosthesis user and the more realistic the appearance of the device, the more satisfying the aesthetic aspects of the prosthesis were evaluated. Furthermore, male amputees were more satisfied with the aesthetics of the prosthesis than female amputees. This model explained *R*^2^ = 14.3% of variance in aesthetic prosthesis satisfaction. Model VII added prosthesis embodiment to the regressors, and also had a significant determination coefficient of *R*^2^ = 18.6% [*F*_(8,152)_ = 4.353, *p* < 0.001], representing a significant increase compared to the former model [*F*_(1,152)_ = 8.040, *p* = 0.005]. Besides *sex* and *age*, prosthesis embodiment emerged as the only significant regressor, indicating that higher prosthesis embodiment is associated with higher aesthetic prosthesis satisfaction, while concurrently absorbing explanatory power of *visual realism*. The association between the levels of aesthetic prosthesis satisfaction for each item of the respective TAPES-R sub-scale is given in Figure 2A.

Model VIII, regressing on functional prosthesis satisfaction and involving the same set of regressors as in model VI, again had a significant determination coefficient [*F*_(7,153)_ = 4.818, *p* < 0.001], with *RLP intensity* and *mobility* having individual and significant associations with the criterion. This suggests that less residual limb pain and higher functionality are significantly related to a higher degree of functional prosthesis satisfaction. Interestingly, compared to the previous regression on aesthetic prosthesis satisfaction, sex, and age did not emerge as significant regressors in this model, which in total explained *R*^2^ = 18.1% of the criterion's variance. As before, model IX added prosthesis embodiment to the regressors, and again its determination coefficient was characterized by significance [*F*_(8,152)_ = 5.958, *p* < 0.001]. In total, this model explained *R*^2^ = 23.9% of variance in functional prosthesis satisfaction, which again represented a significant increase in the determination coefficient [*F*_(1,152)_ = 11.601, *p* = 0.001]. Besides *RLP intensity*, only *prosthesis embodiment* emerged as a significant regressor for higher functional prosthesis satisfaction, canceling out the explanatory power of *mobility*. The association between the levels of functional prosthesis satisfaction for each item of the respective TAPES-R sub-scale is visualized in Figure 2B.

## Discussion

The present study sought to identify amputation- and prosthesis-related factors significantly associated with prosthesis embodiment experiences in lower limb amputees. A hierarchical regression analytical approach was used, and variables were grouped dependent on their objective-descriptive (e.g., amputation level or time with current prosthesis) or subjective-evaluative character (e.g., residual limb pain intensity or rated mobility). Entering significant regressors in a combined regression model revealed that a lower level of amputation, less severe residual limb pain, more realistic visual appearance of the device, higher mobility, and more positive valence of prosthesis-induced residual limb stimulations individually and significantly explained variance in prosthesis embodiment. Together with demographic variables, this model explained more than 1/3 of prosthesis embodiment variance in the present sample. Using the identical set of regressors, hierarchically complemented by prosthesis embodiment, on different forms of prosthesis satisfaction revealed that prosthesis embodiment adds a significant amount of explanatory power to models on both aesthetic and functional prosthesis satisfaction. These findings emphasize the importance of psychological factors for the integration of a prosthesis into the amputee's body representation, which itself might represent a crucial factor associated with prosthesis satisfaction.

These results might be of crucial interest for shaping theoretical concepts on prosthesis embodiment as well as for prosthetic rehabilitation. Thus, the results add to the large body of literature investigating the mechanisms underlying embodiment experiences, since Botvinick and Cohen (1998) introduced the rubber hand illusion paradigm. This seminal experiment involves a setup to induce embodiment experiences (particularly *Ownership, Agency*, and spatial-structural aspects referred to as *Location*; Longo et al., 2008) for a visible artificial limb in non-amputated individuals by applying synchronous visuotactile stimulation to the rubber hand and the participant's hidden hand. Since even upper limb amputees can be induced to perceive the rubber hand illusion—by the application of visuotactile stimulation to an artificial hand together with the amputee's residual limb (Ehrsson et al., 2008)—it has been proposed that this kind of illusion could be an experimental model for prosthesis embodiment as well (Giummarra et al., 2008; Niedernhuber et al., 2018). Empirical evidence for psychometric similarity between non-amputated individuals experiencing the rubber hand illusion and lower limb amputees using a prosthesis was recently provided (Bekrater-Bodmann, 2020). Contrary to the rubber hand illusion, in which multimodal sensory input leads to the integration of the artificial limb into the amputees body representation, prosthesis embodiment could be achieved by sensorimotor (cf., Kalckert and Ehrsson, 2012) or phantom-prosthesis interactions (Giummarra et al., 2010), although the actual processes still remain unknown. The present study, however, describes factors that extend beyond active sensory feedback and neural-machine-interfaces, which are the main foci of current prosthesis embodiment literature (e.g., Marasco et al., 2011; Tabot et al., 2013; Clites et al., 2018; Petrini et al., 2019; Rognini et al., 2019), potentially guiding future prosthetic developments.

Results suggest that objective-descriptive amputation- and prosthesis-related variables can only explain a small amount of the variance in prosthesis embodiment in the present sample. Entering subjective-evaluative variables significantly enhanced the explanatory power of the models. The combination of significant regressors explained the greatest amount of variance in prosthesis embodiment (*R*^2^ = 36.3%). Thus, the level of amputation emerged as a significant regressor in this model which might be indicative of higher perceptual barriers associated with more severe limb loss. This could be due to the longer prosthesis which has to be incorporated in higher amputation levels. A longer prosthetic device might represent a mismatch to the body representation which hinders its incorporation. Previous results suggested that there are distortions of the peripersonal space representation around the residual limb of amputees (Canzoneri et al., 2013). The peripersonal space, however, marks the barrier within which the induction of embodiment experiences is successful (Lloyd, 2007). Consequently, for prosthetic limbs outside these representational boundaries (which is about 70 cm for the lower limbs; Stone et al., 2018) the experience of embodiment might be reduced.

Results further indicate that residual limb pain is negatively associated with prosthesis embodiment. Together with the like-wise significant, but individual, regressor *residual limb stimulation*, this is an interesting finding which emphasizes the importance stump health and proper fit of the device might have for eliciting prosthesis embodiment experiences. On a psychological level, adverse stump experiences, which can be exaggerated by bad prosthesis fit, might reduce the acceptance of the device and thus its embodiment. Previous studies revealed the relevance of socket properties for prosthesis satisfaction (Ali et al., 2012), so that prospective studies should focus on its importance for prosthesis embodiment as well.

Mobility emerged as significant regressor for prosthesis embodiment in the present study, emphasizing the importance of prosthesis functionality for the incorporation of the device. Imaizumi et al. (2016) argued that motor learning and subsequent internal body model updates are consequences of long-term prosthesis use and thus contribute to prosthesis embodiment. However, since neither *time since amputation* nor *time with current prosthesis* were significantly associated with prosthesis embodiment, and *frequency of prosthesis use* only emerged as a significant regressor when *mobility* was not included, the results suggest that the quality of prosthesis use, rather than passive or active use alone, is crucial for inducing embodiment experiences. However, it has to be kept in mind that the present study only assessed the subjective evaluation of mobility; prospective studies should substantiate this finding by implementing objectifiable measures of prosthetic function and the quality of its use. In this context, it might be particularly interesting to further elucidate the satisfaction with usefulness of the device (see Figure 2) which showed particularly strong associations with prosthesis embodiment.

The positive correlation between perceived visual realism of the prosthesis and its embodiment suggests that prosthesis appearance might play a role for incorporation of the device. A similar effect has been previously shown for the experimental induction of embodiment (Tsakiris et al., 2010). It is remarkable, however, that this effect plays a role for lower limb prostheses, whose users—compared to users of upper limb prostheses—are less often directly faced with the device. It could be that prosthetists therefore implicitly assume that realism is of secondary importance which would explain the often-implemented technical appearance of lower limb prostheses. The present results, however, suggest that prostheses that are aesthetically designed in accordance to the user's body perception might facilitate its embodiment. This might particularly be true for the shape of the prosthesis resembling a real limb, since satisfaction with this feature seems to be specifically associated with prosthesis embodiment (see Figure 2). The shape of the prosthesis resembling a real limb could be of particular importance for amputees who habitually have an unfavorably low manifestation of the perceptual trait underlying embodiment experiences (cf., Bekrater-Bodmann et al., 2012). More research is required to elucidate the importance of visual prosthesis characteristics and its interaction with the user's embodiment experiences.

Interestingly, prosthesis embodiment emerged as an important factor associated with both aesthetic and functional prosthesis satisfaction, independently of the other identified variables. This is an extension of findings reported before: using a sub-sample of the present one, the univariate relationship (as revealed by Spearman correlations) between prosthesis satisfaction and prosthesis embodiment has already been reported (Bekrater-Bodmann, 2020). However, the present multivariate analytical approach substantiates this finding, emphasizing that prosthesis embodiment significantly contributes to prosthesis satisfaction even if the association of other relevant factors is statistically controlled for. This emphasizes the relevance prosthesis embodiment might have for prosthesis acceptance. Besides prosthesis embodiment, only sex and age emerged as significant regressors for aesthetic prosthesis satisfaction, probably emphasizing the technical affinity in younger and male persons (Edison and Geissler, 2003). The analyses for functional prosthesis satisfaction further revealed the importance of residual limb pain, supporting previous results (Baars et al., 2018). Thus, although prosthesis embodiment appeared to explain a substantial amount of both aesthetic and functional prosthesis satisfaction, demographic and medical conditions should also be taken into account as moderating variables. It is remarkable, however, that the relationship between prosthetic feature satisfaction and prosthesis embodiment was found for each item of the TAPES-R prosthesis satisfaction sub-scales (Figure 2). Besides classical aesthetic and functional features, satisfaction with the device's weight might be of particular importance (Sinha et al., 2014), since it directly relates to the constructional design of the prosthesis. The relationship between single features of the prosthesis and its embodiment might thus be of crucial interest for prosthesis developers.

The findings indicate that the interaction between body and prosthesis perception should be considered in addition to cosmetic and functional aspects of the prosthesis. The identification of perceptual deficits related to the prosthesis at an early stage might help to fix user problems which might be easily overlooked otherwise. Further, the literature on embodiment experiences in normally-limbed persons suggest that incorporation of an artificial body part into one's body representation can be facilitated by reducing multimodal sensory or sensorimotor conflicts in relation to a cortically stored body model (for reviews see Tsakiris, 2010; Riemer et al., 2019). For limb amputees, characterized by an altered body representation, these factors might even increase in importance. The present results emphasize that a prosthesis which successfully interacts with the user's body perception could enhance prosthesis acceptance and thus reduce the risk of prosthesis abandonment. Moreover, recent advances suggest that sensory feedback might further enhance lower limb prosthesis embodiment (Petrini et al., 2019), which has been earlier reported also for upper limb prostheses (Rognini et al., 2019). How these technical innovations might interfere with the factors identified in the present study, however, remains unknown.

It has to be noted that some of the present results (e.g., the positive association between the level of amputation and prosthesis embodiment) support previous studies, while others do not (e.g., the non-significant association between time since amputation and prosthesis embodiment; cf., Bekrater-Bodmann et al., 2020). This might be due to the fact that the earlier study assessed only a subcomponent of prosthesis embodiment (i.e., ownership; cf., Longo et al., 2008), while the PEmbS-LLA assesses prosthesis embodiment multidimensionally. The differences in sample size might further cause different levels of statistical power. Future studies have to evaluate the differential relationships to other components of prosthesis embodiment (Murray, 2008; Makin et al., 2017).

There are several limitations of the present study. Thus, regression analyses can identify associations, but cannot reveal causal relationships. For instance, it remains open whether visual realism of the prosthesis enhances its embodiment, or whether embodiment experiences lead to perceived similarity of the prosthesis to an actual body part (cf., Longo et al., 2009). Likewise, prosthesis embodiment could lead to satisfaction with the device, but it could also be the other way around, that is, satisfaction could cause the device's incorporation. For other objective-descriptive characteristics, such as the level of amputation, the direction of relationship appears clearer. It is likely that there are complex interactions between different variables. Prospective experimental studies, manipulating identified factors by keeping others constant, are necessary to answer the question of causality. These studies should also systematically compare technical properties of the prosthesis. For instance, recent results indicate that rather naturalistic designs might be associated with higher prosthesis embodiment (Bekrater-Bodmann et al., 2020), and other results emphasize the importance of socket liner characteristics for prosthesis satisfaction (Ali et al., 2012).

In general, the results of regression analyses highly depend on the entered variables. The selection of regressors in the present study was guided by previous findings and theoretical considerations on prosthesis embodiment; however, technical properties of the prosthesis were excluded. Thus, the present results have to be seen as starting point for prospective studies on prosthetic properties and their impact on prosthesis embodiment. These studies could elucidate the large amount of unexplained prosthesis embodiment variance, which was nearly 2/3 in the present study. A recent study estimated the effects of prosthetic features on embodiment experiences at about 40%; endogenous constraints, in terms of relatively stable perceptual traits related to the degree of flexibility of the body representation system, might account for another 30% of the unexplained variance (Bekrater-Bodmann, 2020). The quantification of effects of intra-individually invariant characteristics and external open-to-influence features has to be performed by future studies.

Further, it has to be noted that prosthesis-using participants might represent a particular sample of lower limb amputees. Most of the participants in the present study lost their leg by accidents, which is different to the general population of lower limb amputees (Moxey et al., 2010) who display a higher percentage of peripheral vascular diseases. Since amputations caused by the latter reason are at a higher risk to develop post-amputation pain (Larbig et al., 2019), the found relationship between RLP (which had a relatively low prevalence; cf., Ehde et al., 2000) and prosthesis embodiment has to be further elucidated in the future. Finally, the present results cannot be generalized to other clinical samples characterized by limb loss, such as arm amputees or persons with congenitally absent limbs, and should also be replicated in an independent sample of lower limb amputees. In those future studies, implicit or behavioral measures should be considered to operationalize the factors identified in the present sample.

## Conclusion

Objective-descriptive and subjective-evaluative factors contribute to the embodiment of a lower limb prosthesis, complementing current technical approaches that focus on the effects of multimodal sensory and sensorimotor feedback. In addition to cosmetic and functional aspects of the prosthesis, prosthesis embodiment has been identified as contributing to the user's satisfaction with the prosthetic device. Future studies have to elucidate the underlying neurocognitive processes in order to translate the findings into practical recommendations for prosthesis developers and professionals working in prosthetic rehabilitation.

## Data Availability Statement

The dataset analyzed for the current study is available from the corresponding author on reasonable request.

## Ethics Statement

The studies involving human participants were reviewed and approved by ethics commission II, Heidelberg University. The patients/participants provided their written informed consent to participate in this study.

## Author Contributions

RB-B wrote the manuscript.

## Conflict of Interest

The author declares that the research was conducted in the absence of any commercial or financial relationships that could be construed as a potential conflict of interest. The handling Editor declared a past co-authorship with the author RB-B.
