# Supplementary material for: Factors Associated With Prosthesis Embodiment and Its Importance for Prosthetic Satisfaction in Lower Limb Amputees
Source: Front Neurorobot. 2021 Jan 15;14:604376. doi: 10.3389/fnbot.2020.604376 (PMC7843383; doi:10.3389/fnbot.2020.604376)
Supplement: Supplementary file 1 [file Table_1.DOCX]

Factors associated with prosthesis embodiment and its importance for prosthetic satisfaction in lower limb amputees

Robin Bekrater-Bodmann

**– Supplement –**

**Test of statistical assumptions for regression analyses**

#### Models I and II

For the hierarchical regression analysis on prosthesis embodiment simultaneously entering objective-descriptive (model I) and objective-descriptive + subjective-evaluative amputation-related predictors (model II), there was no evidence for deviation from normal distribution of the residuals (*W*_161_ = .992, *p* = .522), multicollinearity (tolerances between .721 and .960, *VIF* between 1.041 and 1.386), heteroscedasticity (*χ*^2^_1_ = .003, *p* = .954), autocorrelation of residuals, or endogeneity.

#### Models III and IV

For the hierarchical regression analysis on prosthesis embodiment simultaneously entering objective-descriptive (model III) and objective-descriptive + subjective-evaluative prosthesis-related predictors (model IV), there was no evidence for a deviation from the residual’s normal distribution (*W*_159_ = .993, *p* = .635), multicollinearity (tolerances between .942 and .998, *VIF* between 1.002 and 1.062), heteroscedasticity (*χ*^2^_1_ = .015, *p* = .904), autocorrelation of residuals, or endogeneity, so that all assumptions were fulfilled.

**Model V**

For the combined regression analysis, simultaneously entering the regressors identified to individually explain prosthesis embodiment in the previous analyses (i.e., *level of amputation*, *RLP intensity*, *visual realism*, *mobility*, and *residual limb stimulation*), together with *sex* and *age* (model V), there was no evidence for non-normal distribution of residuals (*W*_161_ = .998, *p* = .996), multicollinearity (tolerances between .765 and .945, *VIF* between 1.058 and 1.308), heteroscedasticity (*χ*^2^_1_ = .085, *p* = .770), autocorrelation of residuals, or endogeneity.

**Models VI-IX**

For the hierarchical regression analyses on aesthetic and functional prosthesis satisfaction (, the regressors used in model V were entered (first hierarchical level, models VI and VIII) and complemented by prosthesis embodiment (second hierarchical level, models VII and IX). There was no evidence for deviation from normal distribution of the residuals (*W*_161_ = .987, *p* = .145 for aesthetic prosthesis satisfaction, and *W*_161_ = .989, *p* = .224 for functional prosthesis satisfaction), multicollinearity (tolerances between .637 and .945 and *VIF* between 1.058 and 1.569), heteroscedasticity (*χ*^2^_1_ = .267, *p* = .605 for aesthetic prosthesis satisfaction, and *χ*^2^_1_ = .010, *p* = .920 for functional prosthesis satisfaction), autocorrelation of residuals, or endogeneity.

Table S1: Overview of items and included variables

| **Item wording** | **Response scale(s)** | **Variables and its definitions** |
| --- | --- | --- |
| **Objective-descriptive amputation-related factors** | | |
| *Please indicate the date of your amputation.* | - dd/mm/yy | *Time since amputation*: Difference between amputation and study participation (in full years) |
| *What type of amputation do you have?* | - Foot amputation - Transtibial amputation - Knee exarticulation - Transfemoral amputation - Hip exarticulation or hemipelvectomy | *Level of amputation*:  Dichotomization of responses; low amputation level (0) = foot and transtibial amputation; high amputation level (1) = knee exarticulation, transfemoral amputation, and hip exarticulation or hemipelvectomy |
| *What was the reason for your amputation? (multiple responses allowed)* | - Accident - Injury - Cancer - Infection - Peripheral vascular disease - Congenital limb deficiency - Other reasons | *Etiology of amputation*:  Dichotomization of responses; traumatic events (0) = accidents and injuries; other events (1) = other or multiple reasons |
| **Subjective-evaluative amputation-related factors** | | |
| *The following questions refer to phantom limb awareness, that is, the awareness of the body part that is no longer present. Have you been aware of a phantom limb in the last three months?* | - No, I have never been aware of a phantom limb - No, I have not been aware of a phantom limb in the last three months, but I did so in the past - Yes, I was aware of a phantom limb | *Phantom limb awareness intensity*  (If a participant stated that he or she have not been aware of a phantom limb in the last three months, an intensity measure of 0 was entered) |
| *If yes: Please specify your average phantom limb awareness intensity in the last four weeks.* | Numerical rating scale from 0 (no sensations) to 10 (very strong sensations) |  |
| *The following questions refer to phantom limb pain, that is, painful sensations in the body part that is no longer present. Have you experienced phantom limb pain in the last three months?* | - No, I have never experienced phantom limb pain - No, I have not experienced phantom limb pain in the last three months, but I did so in the past - Yes, experienced phantom limb pain | *Phantom limb pain intensity*  (If a participant stated that he or she have not experienced phantom limb pain in the last three months, an intensity measure of 0 was entered) |
| *If yes: Please specify your average phantom limb pain intensity in the last four weeks.* | Numerical rating scale from 0 (no pain) to 10 (unbearable pain) |  |
| *The following questions refer to residual limb pain, that is, painful sensations in the stump of the amputated body part. Have you experienced residual limb pain in the last three months?* | - No, I have never experienced residual limb pain - No, I have not experienced residual limb pain in the last three months, but I did so in the past - Yes, experienced residual limb pain | *Residual limb pain intensity*  (If a participant stated that he or she have not experienced phantom limb pain in the last three months, an intensity measure of 0 was entered) |
| *If yes: Please specify your average residual limb pain intensity in the last four weeks.* | Numerical rating scale from 0 (no pain) to 10 (unbearable pain) |  |
| **Objective-descriptive prosthesis-related factors** | | |
| *Since when do you use your current prosthesis?* | mm/yy | *Time with current prosthesis*: Difference between indicated date and study participation (in full years) |
| *How often do you use your prosthesis?* | a) per week   - 1 - less than twice - 2 - every second day - 3 - almost daily - 4 - daily   b) per day   - 1 - one to two hours - 2 - several hours, but not throughout - 3 - half a day - 4 - from morning to evening | *Frequency of prosthesis use*:  Ratings per week (1, 2, 3, 4) × per day (1, 2, 3, 4), converted into 9 ranks |
| **Subjective-evaluative prosthesis-related factors** | | |
| *Please evaluate the visual appearance of your prosthesis.* | Numerical rating scale from 0 (artificial) to 10 (like an actual body part) | *Visual realism* |
| PLUS-M-12 as telephone interview (Hafner et al., 2017): 12 items targeting the perceived ability to perform everyday actions; response scale ranging from 0 (without any difficulty) to 4 (unable to do) | | *Mobility*:  Raw scores were converted to T-values (reversed) according to the guidelines (see main text for details) |
| *Is the stump stimulated by the prosthesis?* | - yes - no | *Residual limb stimulation*: negative ratings were recoded to -1, neutral (i.e., pleasantness/unpleasantness of 0) or absent stimulations to 0, and positive ratings to +1 |
| *If yes: How pleasant / unpleasant are these stimulations on average?* | Likert scale ranging from -5 (very unpleasant) to +5 (very pleasant). |  |

Reference for the PLUS-M-12: Hafner, B. J., Gaunaurd, I. A., Morgan, S. J., Amtmann, D., Salem, R., and Gailey, R. S. (2017). Construct Validity of the Prosthetic Limb Users Survey of Mobility (PLUS-M) in Adults with Lower Limb Amputation. Archives of Physical Medicine and Rehabilitation 98, 277–285. doi:10.1016/j.apmr.2016.07.026.
